# Exosomes and miRNA-Loaded Biomimetic Nanovehicles, a Focus on Their Potentials Preventing Type-2 Diabetes Linked to Metabolic Syndrome

**DOI:** 10.3389/fimmu.2018.02711

**Published:** 2018-11-21

**Authors:** Diane Beuzelin, Bertrand Kaeffer

**Affiliations:** UMR-1280 Inra and University of Nantes, Nantes, France

**Keywords:** nanoparticle, epigenetics, metabolic diseases, neonate, breast milk

## Abstract

Exosomes are small membrane vesicles of 30–150 nm, members of the extracellular vesicle family and secreted by various cell types. Different studies describe specific microRNA (miRNA) with altered expression in serum and/or plasma of patients suffering from diabetes or metabolic syndrome. Diabetic cardiomyocyte-derived exosomes loaded with miRNAs like miR-320-3p (or 320a) have been shown regulating angiogenesis on endothelial cell cultures. Insufficient myocardial angiogenesis is the major manifestation of diabetes-caused ischemic cardiovascular disease. Studies on transfer of functional microRNAs between mouse dendritic cells via exosomes have shown that some miRNAs (miR-320-3p, 29b-3p, 7a-5p) are distributed in immature and mature exosomes. Among these miRNAs, miR-320-3p is better known in epigenetics for silencing polr3d gene by binding to its promoter in Human Embryonic Kidney-293 cells. Moreover, quantitative and stoichiometric analysis of the microRNA content of exosomes highlights the lack of reliable natural source of such particles loaded with miRNA opening the need for tailoring exosomes or nanoparticles delivering efficiently miRNA intimately linked to immunity, metabolism and epigenetics in target cells. However, loading of extracellular mature miRNA into recipient cells comes with a cost by at least impeding dynamic localization of miRNAs in nucleoli or inefficient miRNA delivery due to rapid recycling by exonucleases. All these works are calling for the design of new biomimetic vehicles and *in vivo* assessment of miRNA functionality when delivered by natural or biomimetic nanoparticles in order to control metabolic diseases from infancy to adulthood.

## Introduction

Exosomes are nano-vesicles naturally released from living cells, mediating cell-to-cell communication, possibly by delivering RNA cargo (1). Some exosomes deliver functional mature mRNA during inflammation from hematopoietic system to brain (2). Furthermore, human trophoblast BeWo cell line in culture express and secrete placenta-specific miRNAs (miR-517a) in exosomes suggesting their secretion into maternal circulation (3). Stevanato et al. (4) have quantified by real-time PCR a highly shuttled exosomal miRNA subtype (hsa-miR-1246) in tumoral exosomes, despite a negative report that raised concern about the value of these extracellular vesicles (EVs) as shuttle of exogenous miRNAs (5). Exosomes or biomimetic nanovesicles would be useful in preventative or curative strategies of type-2 diabetes (T2D) and related metabolic syndrome. Diabetes mellitus is a group of metabolic diseases characterized by high blood glucose either due to lack of (type 1) or resistance to (type 2) insulin. MiRNAs are intimately linked to immunity and metabolism: several studies describe specific miRNA with altered expression in serum and/or plasma of patients with metabolic syndrome and diabetes. Among 59 independent studies (6), 158 miRNAs are dysregulated in seven different major sample types (adipose, islet, skeletal muscle, whole blood, PBMC, plasma, and serum). For instance, members of the miR-29 family (7), miR-320-3p (or 320a) (8), and miR-7a (9) are notably involved in the regulation of insulin secretion and/or insulin signaling pathways.

This minireview will present current knowledge on loading and unloading of miRNA in exosomes, focusing on proofs obtained with miR-320-3p-loaded exosomes in diabetic context, and on designing biomimetic nanoparticles to transfer efficiently miRNAs.

## Loading miRNA into exosomes

**Figure 1:**
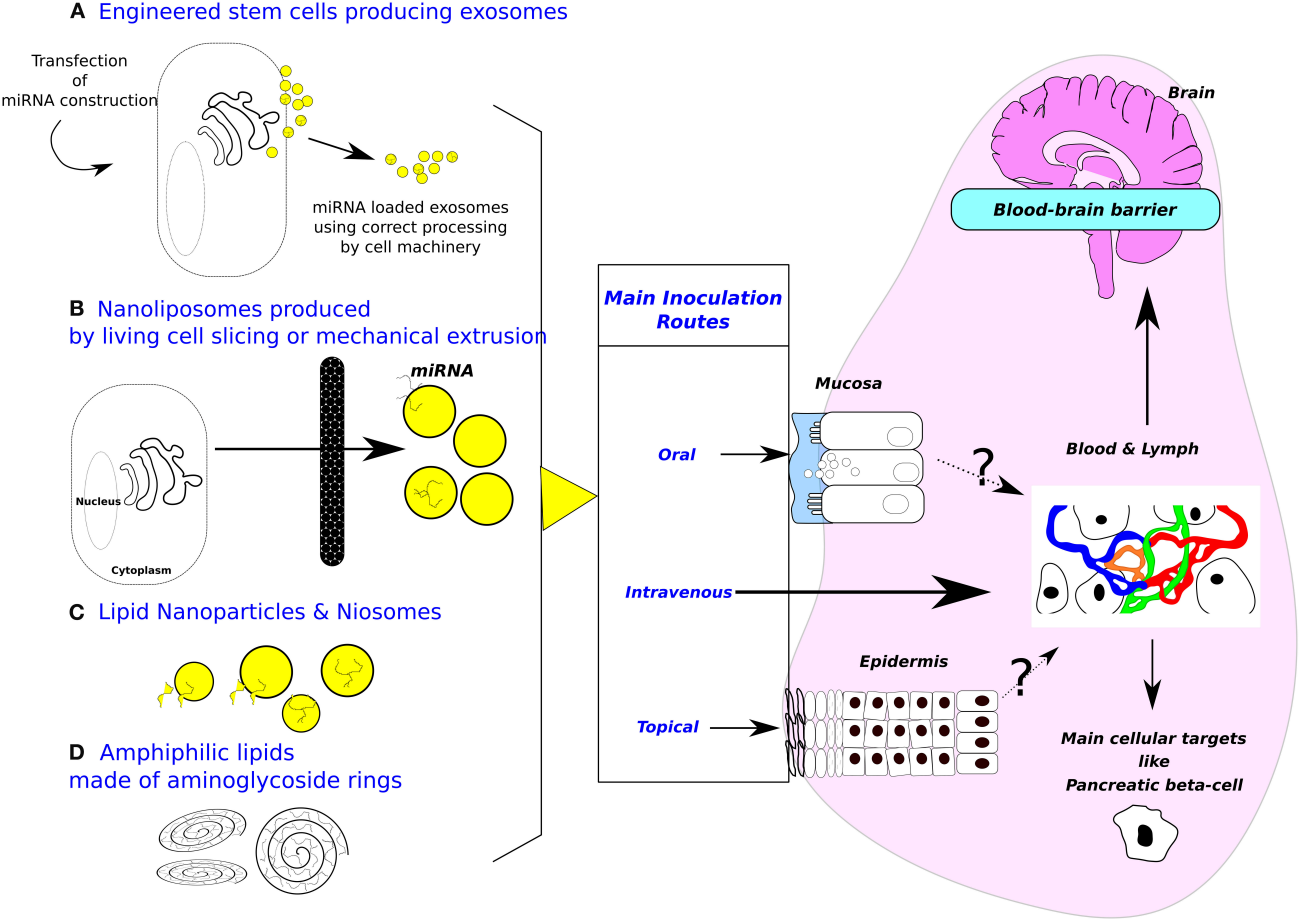
Sources of exosomes and design of biomimetic vehicles. Purposes of inoculation routes are (1) minimizing invasiveness, (2) targeting cell-specific (i. e., gut or beta pancreatic cells) or tissue-specific delivery (i. e., brain). **(A)** Engineered cells for producing exosomes. **(B)** Nanoliposomes produced from living cells. **(C)** Lipid nanoparticles & Niosomes (Diameter between 10–100 nm, made with non-ionic surfactants including alkyl esters, ethers, and amides.). Most applications are targeting T lymphocytes or dendritic cells. Beside these attempts to mimic more or less closely natural exosomes, amphiphilic lipids made of aminoglycosides rings **(D)** are also used to deliver nucleic acid in target cells, the main application being vaccination. Mechanism of cytosol delivery has been studied under electron microscopy (23). Moreover, oral inoculation opens the possibility to target digestive epithelial cells (24). Note that blood-brain barrier at the plexus choroid can relay signal received from circulating exosomes by producing *de novo* exosomes liberated in brain area (25). A cellular target could be pancreatic beta-cells, the cell surface proteome has been described (26) opening the way to specifically address biodesigned nanoparticles loaded with miRNAs. Patients undergoing bariatric surgery are frequently recovering from type-2 diabetes, strongly suggesting that gastrointestinal epithelium is crucial in such pathology and opening the way for treating metabolic diseases by oral delivery of drugs (27).

MiRNAs are short sequences of non-coding RNAs that have emerged as genomic regulators of critical physiological and cellular functions (10). Inside cells, the miRNA precursor is transcribed into a primary pri-miRNA, processed by Rnase-III Drosha. Afterwards this pre-miRNA is transported to the cytoplasm and trimmed by Dicer. The mature miRNA is integrated into a RNA-Induced Silencing Complex comprising Argonaute and, to facilitate the translational repression of target mRNA, the glycine-tryptophan protein of 182 kDa [GW182, also TNRC6A-C in mammals; (11)]. In a monocyte cell line (12), MonoMac-6, some miRNAs like miR-16-5p or let-7a are associated with secreted vesicles (exosomes) that derive from endo-lysosomal compartments called multivesicular bodies (MVBs). In Oli-neu cells, a mouse oligodendroglial cell line that contains a large number of MVBs, the miRNA-Proteolipid protein cargo is segregated into distinct subdomains on the endosomal membrane. The transfer of exosome-associated domains into the lumen of the endosome does not depend on the function of the Endosomal Sorting Complexes Required for Transport machinery, but required the sphingolipid ceramide (13). Localization of miRNAs into exosomes has been related with sequence motifs present in mature miRNAs allowing specific binding by sumoylated protein heterogeneous nuclear ribonucleoprotein A2B1 [hnRNPA2B1; (14)]. Some importation sequences are harbored by miRNAs targeting them as cell resident or exosomal miRNA [GGAG or CCCU; (14)]. However, Chevillet et al. have explored exosome sources of human origin from plasma, seminal fluid, dendritic cells, mast cells, and ovarian cancer cells (5). Overall, they found 0.8% of total exosomes with one copy of miRNA (from 11.1% for miR-720 in seminal fluid down to 0.002% for miR-126 in healthy donor plasma). These results are consistent with a small fraction of exosomes carrying a low concentration of miRNAs, or with the existence of few exosomes in the population highly enriched in specific miRNA. These low-occupancy models need much lower concentrations of miRNA delivery than traditional RNA-induced silencing complex targeting mRNA. It is worth noticing that both low-occupancy models are compatible with recently proposed non-conventional activities of miRNAs. These include the elicitation of cellular responses through binding of Toll-like receptors along with potential effects of small RNAs on DNA transcription and/or epigenetic states. In other words, the mature miRNA can be harbored at the surface of EVs moving the debate from efficient delivery of miRNA in cytoplasm to participation of miRNA to molecular complexes binding target cell-receptors. In biological fluids, miRNAs are mostly present as single-strand mature forms associated or not with proteins of the RISC like Argonaute-2. Proof associating miRNAs with exosome fraction in ultracentrifugation have been obtained on cell supernatants. MiRNAs are found both within and outside of the 16.5 and 120 K centrifugation pellets which contain most of the known cell-derived vesicles (15). Likewise, microRNAs in peripheral blood microvesicles have been isolated from healthy volunteers (16). Currently, three isolation techniques are used to purify exosome populations: differential centrifugation coupled with ultracentrifugation, epithelial cell adhesion molecule immunoaffinity pull-down, and OptiPrep TM density gradient separation. Mateescu et al. have extensively discussed the difficulties isolating EVs-RNA with potential co-purification with lipoprotein complexes containing miRNA, as well as, distinguishing internal from external miRNA in EVs (17). Moreover, the classification of EVs with nanometric size is still in the making (18) indicating that some very small exosomes (< 50 nm corresponding to 10% of total exosomes) can be detected. In the field of Nanolipidoparticles such a low size (< 25 nm) is not impeding small RNA loading and proper delivery (19). However, in complex biological fluids like plasma or milk, the risk of misinterpretation is high with a lot of High Density Lipoprotein able to transfer miRNA (20, 21) or of macromolecular complexes shuttling miRNA (22). Consequently, miRNA may be exposed to nanoparticle surface either artificial or natural, entering cell as passenger of molecular complexes (Figures 1A–D). Direct entry of double-strand-small RNA is mediated by SID-1 receptors (28), but receptors for single-strand-small RNA are still unknown.

## Unloading of exosomal miRNA cargo in target cell

Wiklander et al. have studied biodistribution of EVs isolated from dendritic cells in mice after systemic delivery (29). These EVs labeled by 1,1-dioctadecyl-3,3,3,3-tetramethy-lindotricarbocyanine iodide generally distribute with highest accumulation in the liver, followed by spleen, gastrointestinal-tract and lungs. Results obtained with inoculations of different EVs sources (muscle, bone marrow, or oligodendrocytes) and in cross-species (human, rat, or mouse) show that EVs may retained the same repertoire of surface receptors and extracellular binding proteins than their parental cells for these three species (29).

The exosome membrane is enriched in lipid-rafts, containing cholesterol, sphyngomyelin, glanglioside GM3, and externalized phosphatidylserine, promoting cellular entry. The membrane fusion process involves also CD9 and CD81, both constitutive tetraspanins of the exosome membrane. The characteristics of exosome entry in target cell are reminiscent of effective viral infection rather than synthetic delivery vehicles. Exosomes are seen to surf on filipodia at the surface of target cells (30). At the base of filopodia, exosomes sort into endosomal trafficking circuits that are targeted to scan the endoplasmic reticulum as a possible site of cargo release. A directed transport of exosomes to the endoplasmic reticulum membrane would therefore allow for an efficient entry of exosomal miRNA cargo into the RNAi translation machinery (30). EV-derived miRNAs could act as a general stabilizer of transcription to compensate effects of cellular stress (31). A report of oligodendrocyte-derived EVs increasing resistance of neurons against different types of stress *in vitro* could be a reflection of this function (32). Nevertheless, loading of exogenous mature miRNAs into recipient cells comes with a cost by impeding dynamic localization of miRNAs in nucleoli. As shown by *in situ* hybridization in human Hela cells, miRNAs accumulate in different cellular organelles/compartment (33). For example, miR-320-3p is 3 to 4 times less detected in nucleoli than in cytoplasm (by comparison miR-29a is 4 to 5 less detected), suggesting a minor accumulation, compared to miR-29b which is 14 to 15 less detected in nucleoli than in cytoplasm (like miR-29c, 11 to 12 less detected).

Among foods, milk is considered highly loaded with nanoparticles, some putatively like exosomes, able to transfer nucleic acids (34). In human breast milk, proteomic analysis has shown a wide diversity of molecular composition with 633 new proteins associated with EVs (35). Direct contact of human milk exosomes labeled with Alexa-Fluor-488 with Human Intestinal Epithelial Cells at 30 min and 2 h, showed that around 10% of them have been localized in the nucleus (36). These results suggest that dynamic profile of exosomes loaded with miRNA can vary in response to altered pH and agressive enzymatic environment in the infant gut, although human milk exosomal RNA are believed sheltered inside Evs from external nucleases. The most promising miRNA is miR-22-3p, targeting transcription factor 7, and regulating gluoconeogenesis (37) which makes it a putative therapeutic target to treat insulin resistance and T2D.

## Detection of miRNA in exosomes associated with type-2 diabetes

This minireview focusses on proofs obtained with miR-320-3p-loaded exosomes in diabetic context (Table 1). Along with its interaction with insulin/glycemia pathways, this miRNA is also known to play a crucial role in epigenetics (41) and is not associated with T1D nor Gestational Diabetes Mellitus (46).

**Table 1:** Known molecular targets of miR-320-3p and relation with exosomes in rodent models.

**Experimental model**	**Molecular target**	**Exosome source and purification**	**References**
Cardiomyocyte cultures of Goto-Kakizaki (GK) rats (10–11 weeks of age, male) in parallel to Wistar rats	IGF-I (proangiogenic factor)	Lower level of exosomes loaded with miR-320-3p than cardiomyocytes of GK rat	(38)
Mouse BDM culture transfert in Dendritic Cells	Ets2 (a transcription factor required for endothelial cell survival) Hsp20	In immature and mature exosomes Proof: Importantly, exosome-shuttle miRNAs are functional, because they repress target mRNAs of acceptor Dendritic Cells	(39)
		
KKAy diabetic mice	Diabetic mice treated with JTXK granule show significant up-regulation of miR-320-3p (2.06-fold) and dow-regulation of miR-320-5p (0.47-fold)	N/A	(40)
HEK-293	Induced enrichment of Ago-1 and EZH2 at the Pol-R-3D locus leading to heterochromatinization and transcriptional gene silencing	N/A	(41)
Human Umbilical Vein Endothelial Cells (HUVECs)	Endothelin 1 (ET-1)	N/A	(8)
	Vascular Endothelial Growth Factor (VEGF)	
	Fibronectin (FN)	
Rat under Chronic Mild Stress	Potential involvement in SERT regulation in Mesocortical Circuit and Its Interplay with Serotonin Transporter Define Resilient Rats in the Chronic Mild Stress	N/A	(42)

*The miR-320 family contains 5 members in human (a, b, c, d, e) all from 3p. Nomenclature is somewhat confusing. The hsa-miR-320-3p is used here because of sequence identity in rat (MIMAT0000903) and mouse (MIMAT0000666). In human it is mainly known as 320a (5′-AAAAGCUGGGUUGAGAGGGCGA-3′; MIMAT0000510). The 18 nucleosides in 5′ are identical to all family and constitute e form; the 3′ end of b is “GCAA,” of c “GU” and of d “A.” This miRNA does not harbor hexanucleotide element putatively facilitating addressing to nucleus (43). In addition, miR-320-5p has been reported only in diabetic mouse pancreatic tissue (40), no report in human. In rat, miR-320-3p is involved in embryo implantation (44) and in mouse is targeting heat-shock protein-20 (also hspb6) in cardiac ischemia/reperfusion injury (45)*.

Early in the course of diabetes, high glucose levels in the bloodstream can lead to endothelial dysfunction and microvascular rarefaction. Insufficient myocardial angiogenesis is the major manifestation of diabetes-caused ischemic cardiovascular disease. Given that diabetic hearts exhibit insufficient angiogenesis, it is significant to test whether diabetic cardiomyocyte-derived exosomes possess any capacity in angiogenesis regulation. Wang et al. have shown that under T2D, significantly higher fluxes of exosomes loaded miR-320-3p have been detected in cardiac cell culture (38). Montecalvo et al. have found that only 5 miRNAs are associated with immature exosomes, 139 miRNAs are associated both with immature and mature exosomes, and 58 are only associated with mature exosomes (39). Whereas, immature dendritic cells down-regulate T-cell responses, mature dendritic cells promote activation, proliferation, and differentiation of effector T cells. Exosomes released by dendritic cells with synchronized maturation were purified from supernatants of Bone Marrow dendritic Cell cultures, either maturation resistant (treated with vitamin D3) or fully mature (LPS-treated). Mature exosomes expressed more CD86 and CD54 and exhibited superior T-cell allostimulatory ability than immature exosomes. The miR-320-3p, along with miR-29a to c, are found both in immature and mature exosomes of Bone Marrow Dendritic Cell cultures. Those exosomes fuse with dendritic cell membranes and transfer their content into the cytoplasm to regulate key dendritic cell functions (39). Live dendritic cells release a large range of EVs, which are partially separated by their pelleting properties [crude separation by filtration and ultracentrifugation; (20)], conditions similar to Montecalvo et al. (39). EVs are heterogeneous and their typology is not related to miRNA loading or any biological functions. Beyond cultured cells like in exosomes of MC/9 mast cells (1), mature hsa-miR-320-3p has been also detected in breast milk exosomes by RNA sequencing (47) and q-PCR (48) as well as in gastric fluid of preterm infants (49).

Moreover, in peripheral blood microvesicles, among 104 miRNAs in EVs, miR-320-3p is highly expressed in microvesicles of plasma (2.637-fold more than in blood cells), as well as, in platelets of healthy human [by comparison miR-29a is 40.31-fold more expressed in blood cells; (16)]. MiR-320-3p has also been associated with the regulation of Glucose-Induced Gene expression in diabetes (8). Likewise, hsa-miR-320-3p is patented as diabetes biomarker (50) and its properties as therapeutic molecules for T2D are partly evaluated. Table 1 shows known miR-320-3p molecular targets and its association with exosomes.

The non-genetic transfer of phenotypic information is considered to involve epigenetic means. Among these, we found DNA methylation, histone modifications, trans-acting and non-nuclear factors like small RNA. The miR-320-3p induces transcriptomic silencing of polr3d in human embryonic kidney 293 cells (41). The polr3d is a gene involved in tumorigenesis (51), coding for the subunit-17 of pol-III (also BN51) associated with cell cycle control (52).

Moreover, some importation sequences are harbored by miRNAs, targeting them as cell resident or exosomal miRNA (14). The miR-320-3p shows a weak Exo-motif as GGCG at the 3′ end, instead of the canonical GGAG or CCCU motifs. In line with this observation, hsa-miR-320-3p is not highly released in exosomes produced by MCF-7 breast cancer cells (53).

However, the main problem related to the use of miR-320-3p in therapy is its multiple targets (Table 1). The miR-320-3p has a strong seeding site in 3′-UTR of hsp20 mRNA in mouse (45), but not in human, and in polr3d promoter for rodents and human (41). This multiple targeting property, shared by many miRNAs, is impeding progress in designing exosome-loaded miRNA therapy. Diabetes treatment that is successful in rodents is using antagomiRs of miR-103/107 (54).

### Perspectives: transferring miRNA with natural or biomimetic nanoparticles

The easiest way to obtain miRNA-loaded exosomes would be from a natural source either cultured cells engineered to produce miRNA packaged in exosomes or a natural product containing high amount of homogeneously loaded exosomes. Sutaria et al. (55) have used HEK293/HEK293T cells to produce pre-miR-199a in high amount of EVs but with inefficient miRNA mimic delivery (Figure 1A). The maximum amount of pre-miR-199a, recovered by ultracentrifugation, is detected at 3 h with levels decreasing at the 6 and 12 h time points (55). These last data seem limiting the use of natural EVs as cargo for miRNA probably because exogenous miRNA are rapidly recycled by XRN1 exonuclease (56). However, the sorting circuit of exosomes has been measured as a stop-and-go movement with peak velocities reaching 8 μm/s in human primary fibroblasts using HEK293 CD63-emGFP exosomes (30). This highly efficient delivery is susceptible to work even under the low occupation model (5). Origin of cell line and clonality may be parameters to consider for future improvements. Embryonic Stem cells are also engineered for enhanced cell proliferation (57) or biofunctionalized liposome-like nanovesicles targeting tumor cells (58). Another possible source of miRNA packaged exosomes is breast milk. If deriving an exosome population from bovine milk has been reported (59), there is no system to obtain an exosome population homogeneously loaded with a single or a cocktail of miRNAs. In addition, it is a current hotly debated issue whether food-derived extracellular miRNAs could cross the gut wall and influence consumer's physiology (34, 36, 60, 61). Milk exosomes loaded with miRNA are able to fuse *in vitro* with human intestinal cell plasma membrane (36) but the delivered miRNAs are diverse and functionality is difficult to prove. An additional difficulty is that the recovery of EVs is influenced by sample collection and vesicle isolation procedure. Fresh samples have to be immediately centrifuged to remove exfoliated cells (62). This is a limitation calling for improved cryopreservation process.

The alternative is nano-sized particles designed with a minimal composition mimicking more or less closely the properties of natural nanoparticles but retaining the desired function, the capacity to deliver bioactive miRNAs. Single-strand-RNAs below 30 bases mimicking mature miRNA are simply by-passing the molecular machinery of duplex pre-miRNA (63). Consequently, biodesigning of nanoparticles mimicking EVs can be reduced to tailoring the envelope and loading with single-strand-RNA. As a general rule documented *in vitro* (64), we need delivering at least 100 molecules in cytoplasm, for bioactivity. These authors found a 5 to 7-fold lower amount of miR-16-5p compared to miR-21-5p with different cellular localization and half-live over 72 h.

Synthetic exosome biomimetic particles are often produced in two-steps. Lipids are dried as film, then hydrated by aqueous medium loaded with the compounds to be encapsulated (65). Man-made exosomes have been produced by mechanical extrusion of living monocytes or macrophages (66) or by coating liposome with cell surface proteins [(67); Figure 1B) and a Ni^2+^-(N-5-amino-1-carboxypentyl)-iminodiacetic acid–containing liposomal system tagged with APO2L/TRAIL (68). The incorporation of certain ligands induces apoptosis and downregulates T cell activation in autoimmune diseases, such as antigen-induced arthritis (68). Another technological implementation is to produce highly immunogenic dendritic-cell derived exosomes (69). These nanovesicles have also been evaluated for the treatment of hematological tumor cells [(70); Figure 1C). Future research promoting exosomes as biovehicles have to clarify apparent contradiction of an exosome production depending on physiopathology status of the patient (2) with identity of surface properties across species (29).

Microfluidic fabrication of cell-derived nanovesicles as endogenous RNA carriers (71) and generation of nanovesicles with sliced cellular membrane fragments for exogenous material delivery have been designed (72). A method based on microemulsification and micelle assembly was described for encapsulating Bovine Serum Albumin as an artificial exosome mimicking antigen presentation to dendritic cells (73). To encapsulate nucleic acid, cationic lipids display the best efficiency, even if they are more immunogenic than their uncharged counterparts (73). Current research is testing vesicles formulated with non-ionic surfactants called niosomes (Figure 1C). Niosomes constitute an original attempt in the development of man-made exosomes. The main advantages of niosomes are a wide array of starting compounds, low cost, improved physical and chemical stability, and higher biocompatibility (65).

Cationic lipids are the most commonly used synthetic delivery vectors. However, a clear need still exists for better delivery of miRNA molecules to improve their biological activity and especially after oral *in vivo* delivery. A novel class of amphiphilic lipids made of aminoglycosides rings know to interact with A form nucleic acids is very efficient to deliver nucleic acid structures inside cells (Figure 1D). The copolymers enhance the cellular uptake of DNA through a facilitated plasma membrane transport where nucleic acids are entrapped in lamellar structure (74, 75). Under our hands, the vector can be used to deliver synthetic miRNA to digestive cells (24). These works are calling for *in vivo* assessment of miRNA functionality when delivered by natural or biomimetic nanoparticles in order to control metabolic diseases from infancy to adulthood. Indeed, improper nutritional handling of preterm babies is a general health problem in the World (76) leading to the onset of a metabolic syndrome through nutritional programming. Epidemiological analysis (77) suggest that human milk is better than artificial infant formula by allowing appropriate nutritional programming and protecting the baby against diseases of civilization in later life (T2D, obesity, hypertension).

In conclusion, recovery from TD2 of patients undergoing bariatric surgery strongly suggests that gastrointestinal epithelium is crucial, opening the way for treating metabolic diseases by oral delivery of drugs (27). *In vivo* delivery of miRNA embedded in biomimetic nanovehicles to gut epithelial cells can pave the way to design new supplementation for breast-fed baby to correct diet-induced nutritional programing leading to diabetes (78).

## Author contributions

All authors listed have made a substantial, direct and intellectual contribution to the work, and approved it for publication.

### Conflict of interest statement

The authors declare that the research was conducted in the absence of any commercial or financial relationships that could be construed as a potential conflict of interest.

## References

[1] Valadi H Ekstrom K Bossios A Sjostrand M Lee JJ Lotvall JO. Exosome-mediated transfer of mRNAs and microRNAs is a novel mechanism of genetic exchange between cells. Nat Cell Biol. (2007) 9:654–9. 10.1038/ncb1596 17486113

[2] Ridder K Keller S Dams M Rupp AK Schlaudraff J Del Turco D. Extracellular vesicle-mediated transfer of genetic information between the hematopoietic system and the brain in response to inflammation. PLoS Biol. (2014) 12:e1001874. 10.1371/journal.pbio.1001874 24893313 PMC4043485

[3] Luo SS Ishibashi O Ishikawa G Ishikawa T Katayama A Mishima T. Human villous trophoblasts express and secrete placenta-specific microRNAs into maternal circulation via exosomes. Biol Reprod. (2009) 81:717–29. 10.1095/biolreprod.108.075481 19494253

[4] Stevanato L Thanabalasundaram L Vysokov N Sinden JD. Investigation of content, stoichiometry and transfer of miRNA from human neural stem cell line derived exosomes. PLoS ONE (2016) 11:e0146353. 10.1371/journal.pone.0146353 26752061 PMC4713432

[5] Chevillet JR Kang Q Ruf IK Briggs HA Vojtech LN Hughes SM. Quantitative and stoichiometric analysis of the microRNA content of exosomes. Proc Natl Acad Sci USA. (2014) 111:14888–93. 10.1073/pnas.1408301111 25267620 PMC4205618

[6] He Y Ding Y Liang B Lin J Kim TK Yu H. A systematic study of dysregulated MicroRNA in type 2 diabetes mellitus. Int J Mol Sci. (2017) 18:456. 10.3390/ijms18030456 28264477 PMC5372489

[7] Dooley J Garcia-Perez JE Sreenivasan J Schlenner SM Vangoitsenhoven R Papadopoulou AS. The microRNA-29 family dictates the balance between homeostatic and pathological glucose handling in diabetes and obesity. Diabetes (2016) 65:53–61. 10.2337/db15-0770 26696639 PMC4876765

[8] Feng B Chakrabarti S. miR-320 regulates glucose-induced gene expression in diabetes. ISRN Endocrinol. (2012) 2012:549875. 10.5402/2012/549875 22900199 PMC3415085

[9] Latreille M Hausser J Stutzer I Zhang Q Hastoy B Gargani S. MicroRNA-7a regulates pancreatic beta cell function. J Clin Invest. (2014) 124:2722–35. 10.1172/JCI73066 24789908 PMC4038573

[10] Bartel DP. MicroRNAs: target recognition and regulatory functions. Cell (2009) 136:215–33. 10.1016/j.cell.2009.01.002 19167326 PMC3794896

[11] Nakanishi K. Anatomy of RISC: how do small RNAs and chaperones activate Argonaute proteins? Wiley Interdiscip Rev RNA (2016) 7:637–60. 10.1002/wrna.1356 27184117 PMC5084781

[12] Gibbings DJ Ciaudo C Erhardt M Voinnet O. Multivesicular bodies associate with components of miRNA effector complexes and modulate miRNA activity. Nat Cell Biol. (2009) 11:1143–9. 10.1038/ncb1929 19684575

[13] Trajkovic K Hsu C Chiantia S Rajendran L Wenzel D Wieland F. Ceramide triggers budding of exosome vesicles into multivesicular endosomes. Science (2008) 319:1244–7. 10.1126/science.1153124 18309083

[14] Villarroya-Beltri C Gutierrez-Vazquez C Sanchez-Cabo F Perez-Hernandez D Vazquez J Martin-Cofreces N. Sumoylated hnRNPA2B1 controls the sorting of miRNAs into exosomes through binding to specific motifs. Nat Commun. (2013) 4:2980. 10.1038/ncomms3980 24356509 PMC3905700

[15] Wang K Zhang S Weber J Baxter D Galas DJ. Export of microRNAs and microRNA-protective protein by mammalian cells. Nucleic Acids Res. (2010) 38:7248–59. 10.1093/nar/gkq601 20615901 PMC2978372

[16] Hunter MP Ismail N Zhang X Aguda BD Lee EJ Yu L. Detection of microRNA expression in human peripheral blood microvesicles. PLoS ONE (2008) 3:e3694. 10.1371/journal.pone.0003694 19002258 PMC2577891

[17] Mateescu B Kowal EJ van Balkom BW Bartel S Bhattacharyya SN Buzas EI. Obstacles and opportunities in the functional analysis of extracellular vesicle RNA - an ISEV position paper. J Extracell Vesicles (2017) 6:1286095. 10.1080/20013078.2017.1286095 28326170 PMC5345583

[18] Kowal J Arras G Colombo M Jouve M Morath JP Primdal-Bengtson B. Proteomic comparison defines novel markers to characterize heterogeneous populations of extracellular vesicle subtypes. Proc Natl Acad Sci USA. (2016) 113:E968–77. 10.1073/pnas.1521230113 26858453 PMC4776515

[19] Tam YY Chen S Cullis PR. Advances in lipid nanoparticles for siRNA delivery. Pharmaceutics (2013) 5:498–507. 10.3390/pharmaceutics5030498 24300520 PMC3836621

[20] Tabet F Vickers KC Cuesta Torres LF Wiese CB Shoucri BM Lambert G. HDL-transferred microRNA-223 regulates ICAM-1 expression in endothelial cells. Nat Commun. (2014) 5:3292. 10.1038/ncomms4292 24576947 PMC4189962

[21] Vickers KC Palmisano BT Shoucri BM Shamburek RD Remaley AT. MicroRNAs are transported in plasma and delivered to recipient cells by high-density lipoproteins. Nat Cell Biol. (2011) 13:423–33. 10.1038/ncb2210 21423178 PMC3074610

[22] Turchinovich A Weiz L Langheinz A Burwinkel B. Characterization of extracellular circulating microRNA. Nucleic Acids Res. (2011) 39:7223–33. 10.1093/nar/gkr254 21609964 PMC3167594

[23] Le Bihan O Chevre R Mornet S Garnier B Pitard B Lambert O Probing the *in vitro* mechanism of action of cationic lipid/DNA lipoplexes at a nanometric scale. Nucleic Acids Res. (2011) 39:1595–609. 10.1093/nar/gkq921 21078679 PMC3045597

[24] Beuzelin D Pitard B Kaeffer B Testing the Transfer of miRNA by Biologically-Inspired Delivery Vehicles on the Physiology of Baby Cells and their Interactions with Gastric Extracellular Vesicles. Paris: French Society for Extracellular Vesicles (2017).

[25] Balusu S Van Wonterghem E De Rycke R Raemdonck K Stremersch S Gevaert K. Identification of a novel mechanism of blood-brain communication during peripheral inflammation via choroid plexus-derived extracellular vesicles. EMBO Mol Med. (2016) 8:1162–83. 10.15252/emmm.201606271 27596437 PMC5048366

[26] Stutzer I Esterhazy D Stoffel M. The pancreatic beta cell surface proteome. Diabetologia (2012) 55:1877–89. 10.1007/s00125-012-2531-3 22460761 PMC3369137

[27] Rubino F. Medical research: Time to think differently about diabetes. Nature (2016) 533:459–61. 10.1038/533459a 27225102

[28] Shih JD Hunter CP. SID-1 is a dsRNA-selective dsRNA-gated channel. RNA (2011) 17:1057–65. 10.1261/rna.2596511 21474576 PMC3096038

[29] Wiklander OP Nordin JZ O'Loughlin A Gustafsson Y Corso G Mager I. Extracellular vesicle *in vivo* biodistribution is determined by cell source, route of administration and targeting. J Extracell Vesicles (2015) 4:26316. 10.3402/jev.v4.26316 25899407 PMC4405624

[30] Heusermann W Hean J Trojer D Steib E von Bueren S Graff-Meyer A. Exosomes surf on filopodia to enter cells at endocytic hot spots, traffic within endosomes, and are targeted to the ER. J Cell Biol. (2016) 213:173–84. 10.1083/jcb.201506084 27114500 PMC5084269

[31] Schratt G. Fine-tuning neural gene expression with microRNAs. Curr Opin Neurobiol. (2009) 19:213–9. 10.1016/j.conb.2009.05.015 19539460

[32] Fruhbeis C Frohlich D Kuo WP Amphornrat J Thilemann S Saab AS. Neurotransmitter-triggered transfer of exosomes mediates oligodendrocyte-neuron communication. PLoS Biol. (2013) 11:e1001604. 10.1371/journal.pbio.1001604 23874151 PMC3706306

[33] Li ZF Liang YM Lau PN Shen W Wang DK Cheung WT. Dynamic localisation of mature microRNAs in Human nucleoli is influenced by exogenous genetic materials. PLoS ONE (2013) 8:e70869. 10.1371/journal.pone.0070869 23940654 PMC3735495

[34] Melnik BC John SM Schmitz G. Milk is not just food but most likely a genetic transfection system activating mTORC1 signaling for postnatal growth. Nutr J. (2013) 12:103. 10.1186/1475-2891-12-103 23883112 PMC3725179

[35] van Herwijnen MJ Zonneveld MI Goerdayal S Nolte-'t Hoen EN Garssen J Stahl B Comprehensive proteomic analysis of human milk-derived extracellular vesicles unveils a novel functional proteome distinct from other milk components. Mol Cell Proteomics (2016) 15:3412–23. 10.1074/mcp.M116.060426 27601599 PMC5098039

[36] Liao Y Du X Li J Lonnerdal B. Human milk exosomes and their microRNAs survive digestion *in vitro* and are taken up by human intestinal cells. Mol Nutr Food Res. (2017) 61:1700082. 10.1002/mnfr.201700082 28688106

[37] Kaur K Vig S Srivastava R Mishra A Singh VP Srivastava AK. Elevated Hepatic miR-22-3p expression impairs gluconeogenesis by silencing the Wnt-responsive transcription factor Tcf7. Diabetes (2015) 64:3659–69. 10.2337/db14-1924 26193896

[38] Wang X Huang W Liu G Cai W Millard RW Wang Y. Cardiomyocytes mediate anti-angiogenesis in type 2 diabetic rats through the exosomal transfer of miR-320 into endothelial cells. J Mol Cell Cardiol. (2014) 74:139–50. 10.1016/j.yjmcc.2014.05.001 24825548 PMC4120246

[39] Montecalvo A Larregina AT Shufesky WJ Stolz DB Sullivan ML Karlsson JM. Mechanism of transfer of functional microRNAs between mouse dendritic cells via exosomes. Blood (2012) 119:756–66. 10.1182/blood-2011-02-338004 22031862 PMC3265200

[40] Mo FF An T Zhang ZJ Liu YF Liu HX Pan YY. Jiang Tang Xiao Ke granule play an anti-diabetic role in diabetic mice pancreatic tissue by regulating the mRNAs and MicroRNAs associated with PI3K-Akt signaling pathway. Front Pharmacol. (2017) 8:795. 10.3389/fphar.2017.00795 29163176 PMC5671979

[41] Kim DH Saetrom P Snove O Jr Rossi JJ. MicroRNA-directed transcriptional gene silencing in mammalian cells. Proc Natl Acad Sci USA. (2008) 105:16230–5. 10.1073/pnas.0808830105 18852463 PMC2571020

[42] Zurawek D Kusmider M Faron-Gorecka A Gruca P Pabian P Solich J. Reciprocal MicroRNA expression in mesocortical circuit and its interplay with serotonin transporter define resilient rats in the chronic mild stress. Mol Neurobiol. (2017) 54:5741–51. 10.1007/s12035-016-0107-9 27660265 PMC5583278

[43] Hwang HW Wentzel EA Mendell JT. A hexanucleotide element directs microRNA nuclear import. Science (2007) 315:97–100. 10.1126/science.1136235 17204650

[44] Xia HF Jin XH Song PP Cui Y Liu CM Ma X. Temporal and spatial regulation of miR-320 in the uterus during embryo implantation in the rat. Int J Mol Sci. (2010) 11:719–30. 10.3390/ijms11020719 20386663 PMC2852863

[45] Ren XP Wu J Wang X Sartor MA Jones K Qian J. MicroRNA-320 is involved in the regulation of cardiac ischemia/reperfusion injury by targeting heat-shock protein 20. Circulation (2009) 119:2357–66. 10.1161/CIRCULATIONAHA.108.814145 19380620 PMC2746735

[46] Collares CV Evangelista AF Xavier DJ Rassi DM Arns T Foss-Freitas MC. Identifying common and specific microRNAs expressed in peripheral blood mononuclear cell of type 1, type 2, and gestational diabetes mellitus patients. BMC Res Notes (2013) 6:491. 10.1186/1756-0500-6-491 24279768 PMC4222092

[47] Zhou Q Li M Wang X Li Q Wang T Zhu Q. Immune-related MicroRNAs are abundant in breast milk exosomes. Int J Biol Sci. (2012) 8:118–23. 10.7150/ijbs.8.118 22211110 PMC3248653

[48] Lasser C Alikhani VS Ekstrom K Eldh M Paredes PT Bossios A. Human saliva, plasma and breast milk exosomes contain RNA: uptake by macrophages. J Transl Med. (2011) 9:9. 10.1186/1479-5876-9-9 21235781 PMC3033821

[49] Kaeffer B Billard H Boquien CY Gauvard E Drouard A Gournay V Early nutrition: transcriptomic profiling of exfoliated cells, microvesicles and exosomes from breast milk and corresponding gastric fluid aspirate of preterm infant. J Dev Orig Health Dis. (2015) 6(Suppl. 1).

[50] Inventors Mayr Manuel Methods and Means for Predicting or Diagnosing Diabetes or Cardiovascular Disorders Based on Micro RNA Detection. King's College London patent WO 2011154689 A1 (2011).

[51] Huang WJ Li M Jin XH Huang XJ Zhao W Tian XP. Genetic profile and biological implication of PIN2/TRF1-interacting telomerase inhibitor 1 (PinX1) in human cancers: an analysis using The Cancer Genome Atlas. Oncotarget (2017) 8:67241–53. 10.18632/oncotarget.18589 28978030 PMC5620170

[52] Ittmann MM. Cell cycle control of the BN51 cell cycle gene which encodes a subunit of RNA polymerase III. Cell Growth Differ. (1994) 5:783–8. 7947392

[53] Pigati L Yaddanapudi SC Iyengar R Kim DJ Hearn SA Danforth D. Selective release of microRNA species from normal and malignant mammary epithelial cells. PLoS ONE (2010) 5:e13515. 10.1371/journal.pone.0013515 20976003 PMC2958125

[54] Trajkovski M Hausser J Soutschek J Bhat B Akin A Zavolan M. MicroRNAs 103 and 107 regulate insulin sensitivity. Nature (2011) 474:649–53. 10.1038/nature10112 21654750

[55] Sutaria DS Jiang J Elgamal OA Pomeroy SM Badawi M Zhu X. Low active loading of cargo into engineered extracellular vesicles results in inefficient miRNA mimic delivery. J Extracell Vesicles (2017) 6:1333882. 10.1080/20013078.2017.1333882 28717424 PMC5505005

[56] Zangari J Ilie M Rouaud F Signetti L Ohanna M Didier R. Rapid decay of engulfed extracellular miRNA by XRN1 exonuclease promotes transient epithelial-mesenchymal transition. Nucleic Acids Res. (2017) 45:4131–41. 10.1093/nar/gkw1284 27994032 PMC5397191

[57] Jeong D Jo W Yoon J Kim J Gianchandani S Gho YS. Nanovesicles engineered from ES cells for enhanced cell proliferation. Biomaterials (2014) 35:9302–10. 10.1016/j.biomaterials.2014.07.047 25132601

[58] Zhang P Zhang L Qin Z Hua S Guo Z Chu C. Genetically engineered liposome-like nanovesicles as active targeted transport platform. Adv Mater. (2018) 30:1705350. 10.1002/adma.201705350 29280210

[59] Munagala R Aqil F Jeyabalan J Gupta RC. Bovine milk-derived exosomes for drug delivery. Cancer Lett. (2016) 371:48–61. 10.1016/j.canlet.2015.10.020 26604130 PMC4706492

[60] Baier SR Nguyen C Xie F Wood JR Zempleni J. MicroRNAs are absorbed in biologically meaningful amounts from nutritionally relevant doses of cow milk and affect gene expression in peripheral blood mononuclear cells, HEK-293 kidney cell cultures, and mouse livers. J Nutr. (2014) 144:1495–500. 10.3945/jn.114.196436 25122645 PMC4162473

[61] Ma J Wang C Long K Zhang H Zhang J Jin L. Exosomal microRNAs in giant panda (Ailuropoda melanoleuca) breast milk: potential maternal regulators for the development of newborn cubs. Sci Rep. (2017) 7:3507. 10.1038/s41598-017-03707-8 28615713 PMC5471263

[62] Zonneveld MI Brisson AR van Herwijnen MJC Tan S van de Lest CHA Redegeld FA. Recovery of extracellular vesicles from human breast milk is influenced by sample collection and vesicle isolation procedures. J Extracell Vesicles (2014) 3:24215. 10.3402/jev.v3.24215 25206958 PMC4139932

[63] Lima WF Prakash TP Murray HM Kinberger GA Li W Chappell AE. Single-stranded siRNAs activate RNAi in animals. Cell (2012) 150:883–94. 10.1016/j.cell.2012.08.014 22939618

[64] Mullokandov G Baccarini A Ruzo A Jayaprakash AD Tung N Israelow B. High-throughput assessment of microRNA activity and function using microRNA sensor and decoy libraries. Nat Methods (2012) 9:840–6. 10.1038/nmeth.2078 22751203 PMC3518396

[65] Garcia-Manrique P Gutierrez G Blanco-Lopez MC. Fully artificial exosomes: towards new theranostic biomaterials. Trends Biotechnol. (2018) 36:10–4. 10.1016/j.tibtech.2017.10.005 29074309

[66] Jang SC Kim OY Yoon CM Choi DS Roh TY Park J. Bioinspired exosome-mimetic nanovesicles for targeted delivery of chemotherapeutics to malignant tumors. ACS Nano (2013) 7:7698–710. 10.1021/nn402232g 24004438

[67] De La Pena H Madrigal JA Rusakiewicz S Bencsik M Cave GW Selman A Artificial exosomes as tools for basic and clinical immunology. J Immunol Methods (2009) 344:121–32. 10.1016/j.jim.2009.03.011 19345222

[68] Martinez-Lostao L Garcia-Alvarez F Basanez G Alegre-Aguaron E Desportes P Larrad L. Liposome-bound APO2L/TRAIL is an effective treatment in a rabbit model of rheumatoid arthritis. Arthritis Rheum. (2010) 62:2272–82. 10.1002/art.27501 20506326

[69] Viaud S Ploix S Lapierre V Thery C Commere PH Tramalloni D. Updated technology to produce highly immunogenic dendritic cell-derived exosomes of clinical grade: a critical role of interferon-gamma. J Immunother. (2011) 34:65–75. 10.1097/CJI.0b013e3181fe535b 21150714

[70] De Miguel D Basanez G Sanchez D Malo PG Marzo I Larrad L Liposomes decorated with Apo2L/TRAIL overcome chemoresistance of human hematologic tumor cells. Mol Pharm. (2013) 10:893–904. 10.1021/mp300258c 23331277

[71] Jo W Jeong D Kim J Cho S Jang SC Han C. Microfluidic fabrication of cell-derived nanovesicles as endogenous RNA carriers. Lab Chip (2014) 14:1261–9. 10.1039/c3lc50993a 24493004

[72] Yoon J Jo W Jeong D Kim J Jeong H Park J. Generation of nanovesicles with sliced cellular membrane fragments for exogenous material delivery. Biomaterials (2015) 59:12–20. 10.1016/j.biomaterials.2015.04.028 25941997

[73] Li K Chang S Wang Z Zhao X Chen D. A novel micro-emulsion and micelle assembling method to prepare DEC205 monoclonal antibody coupled cationic nanoliposomes for simulating exosomes to target dendritic cells. Int J Pharm. (2015) 491:105–12. 10.1016/j.ijpharm.2015.05.068 26073939

[74] Colombani T Peuziat P Dallet L Haudebourg T Mevel M Berchel M. Self-assembling complexes between binary mixtures of lipids with different linkers and nucleic acids promote universal mRNA, DNA and siRNA delivery. J Control Release (2017) 249:131–42. 10.1016/j.jconrel.2017.01.041 28159514

[75] Habrant D Peuziat P Colombani T Dallet L Gehin J Goudeau E. Design of ionizable lipids to overcome the limiting step of endosomal escape: application in the intracellular delivery of mRNA, DNA, and siRNA. J Med Chem. (2016) 59:3046–62. 10.1021/acs.jmedchem.5b01679 26943260

[76] Harville EW Srinivasan S Chen W Berenson GS. Is the metabolic syndrome a “small baby” syndrome?: the bogalusa heart study. Metab Syndr Relat Disord. (2012) 10:413–21. 10.1089/met.2012.0031 22831273 PMC3546360

[77] Ip S Chung M Raman G Chew P Magula N DeVine D. Breastfeeding and maternal and infant health outcomes in developed countries. Evid Rep Technol Assess (Full Rep). (2007) 1–186. 17764214 PMC4781366

[78] Fais S O'Driscoll L Borras FE Buzas E Camussi G Cappello F. Evidence-Based Clinical Use of Nanoscale Extracellular Vesicles in Nanomedicine. ACS Nano (2016) 10:3886–99. 10.1021/acsnano.5b08015 26978483

